# Multifunctional Nanomaterials for Precision Diagnostics and Drug Delivery: AI-Assisted Biosensing, Barrier-Directed Transport, Stimuli-Responsive Release, and Theranostic Integration

**DOI:** 10.3390/molecules31173098

**Published:** 2026-09-04

**Authors:** Stefano Bellucci

**Affiliations:** 1 Qi S.r.l., Via Monte d’Oro 2/A, 00071 Pomezia, RM, Italy; stefano.bellucci@lnf.infn.it; 2 Ecotec University, Km. 13.5 Samborondón, Samborondón 092302, Ecuador

**Keywords:** multifunctional nanomaterials, drug delivery, biosensors, lipid nanoparticles, graphene, carbon nanotubes, biopolymers, nose-to-brain delivery, nanoporous carriers, stimuli-responsive release, theranostics, machine learning, nanotoxicology, critical quality attributes

## Abstract

Nanomaterials are increasingly expected to do more than transport a payload, yet added complexity is useful only when it resolves a rate-limiting diagnostic, transport, release, or monitoring problem. This review develops a function-first framework for precision diagnostics and drug delivery in which formation and processing are linked to nanoscale structure, material properties, demonstrated function, route-specific evidence, and translational value. The scope includes AI-assisted plasmonic and terahertz biosensing; biopolymer nanoparticles and hydrogel depots; barrier-directed nose-to-brain and systemic delivery; graphene and carbon nanotube interfaces; lipid nanoparticles for nucleic acid packaging and endosomal escape; nanoporous, magnetic, and plasmonic carriers; and closed-loop theranostic systems. A platform is treated as genuinely multifunctional only when at least two deliberately engineered functions are experimentally supported and either act on distinct rate-limiting steps or close a sensing–intervention–monitoring loop. This review therefore distinguishes total loading from bioavailable payload, cellular uptake from productive delivery, imaging labels from intact carrier fate, and nominal stimulus responsiveness from controlled release in response to a physiologically realistic trigger. Recent independent studies are used to broaden comparisons across material classes and to separate proof-of-concept performance from translational evidence. Artificial intelligence is considered in three distinct roles—sensor interpretation, formulation/material optimization, and prediction of in vivo behavior—with external validation and, where a model is intended to guide decisions, prospective testing treated as essential. The resulting framework emphasizes biological identity, route-specific safety, carrier-versus-payload tracking, critical quality attributes, manufacturing reproducibility, and a minimum-evidence roadmap from concept to product.

## 1. Introduction

The boundary between a diagnostic nanomaterial and a drug delivery nanocarrier is becoming progressively less useful. A plasmonic surface can identify a biochemical state and, in a related device, serve as a photothermal actuator. A graphene interface can provide a refractive index-sensitive electromagnetic response, adsorb aromatic drugs, reinforce a hydrogel, or convert light into heat. A magnetic nanoparticle can act as a contrast-generating component, a targeting element, or a trigger for local heating. Biopolymers and lipid assemblies can stabilize cargo while also controlling biological interaction, residence, degradation, and release. The central design question is therefore not whether a material is “diagnostic” or “therapeutic”, but which rate-limiting biological or technological problem its nanoscale properties are intended to solve [1,2].

**Figure 1:**
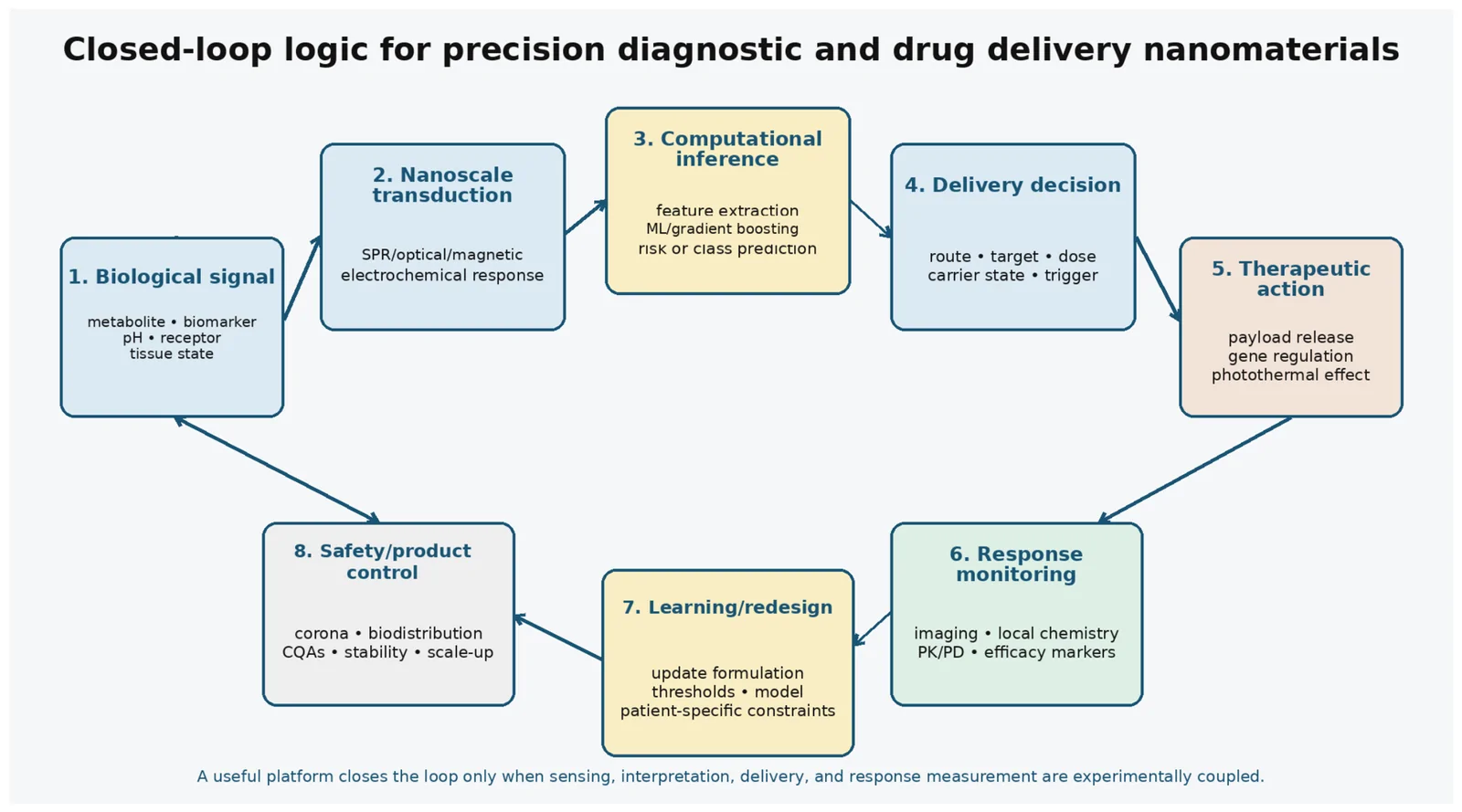
Closed-loop logic for precision diagnostic and drug delivery nanomaterials. The conceptual sequence connects biological sensing to nanoscale transduction, computational inference, delivery or therapeutic action, response monitoring, safety/product control, and iterative redesign. Original schematic prepared for this review.

This function-first view is important because multifunctionality can become an end in itself. Adding a targeting ligand, fluorescent label, magnetic core, responsive linker, polymer shell, or second therapeutic agent may increase the number of measurable features without increasing clinical value. Each additional component introduces its own synthesis tolerance, impurity profile, degradation pathway, analytical method, and biological interaction. In this review, a platform is considered genuinely multifunctional only when at least two deliberately engineered functions are experimentally supported and either address distinct rate-limiting steps or close a sensing–intervention–monitoring loop. Simple coexistence of features is not sufficient. The added function should measurably improve diagnostic discrimination, target site exposure, bioavailable dose, therapeutic outcome, response monitoring, or product control. A credible platform should therefore demonstrate a causal chain: formation or processing creates a defined structure; structure creates a material property; that property changes a measurable intermediate process; and the change improves a biologically or clinically meaningful endpoint. Figure 1 summarizes this closed-loop logic.

This operational boundary also provides an inclusion rule for the material classes discussed below. Graphene and carbon nanotubes are included because geometry, defects, surface chemistry, and electronic structure combine sensing, adsorption, transport, and stimulus response. Biopolymers and hydrogels are included because interfacial chemistry, network structure, and degradation can dominate mucosal or local delivery. Lipid nanoparticles are included because composition and self-assembly control nucleic acid protection, endosomal escape, and organ-level expression and because they provide the clearest current clinical precedent for nanoscale delivery [3,4]. Inorganic, nanoporous, magnetic, and plasmonic materials are included when geometry, accessible pore architecture, magnetic response, or optical response supplies a function that soft carriers cannot easily provide. Material classes are therefore selected to test the framework across distinct mechanisms rather than to provide an exhaustive catalogue.

Representative examples span graphene-based sensing, biopolymer and hydrogel carriers, lipid nanoparticles, carbon nanotubes, and morphology- or porosity-controlled inorganic systems. A 2025 graphene-based terahertz sensor combined dual analyte detection with gradient-boosting prediction, linking engineered electromagnetic response to data-driven interpretation [5], while independent plasmonic and SERS studies demonstrate how machine learning can extract information from experimental sensor images or patient-derived spectra [6,7,8]. Chitosan nanoparticles and hybrid nanoparticle–hydrogel depots illustrate how polymer chemistry can control mucoadhesion, local retention, mechanics, and release [9,10,11]. Intranasal systems provide a barrier-defined test of whether formulation changes actual brain exposure [12,13,14,15]. Lipid nanoparticles extend the design space to nucleic acid protection, endosomal escape, organ targeting, and clinically relevant manufacturing [3,4,16,17,18,19,20]. Carbon nanotubes and graphene systems remain useful mechanistic cases for geometry, functionalization, loading, and toxicology [21,22,23,24,25,26,27,28,29,30,31,32,33], while nanoporous and morphology-controlled inorganic systems show how accessible surface and pore architecture can dominate loading and release [34,35,36,37,38].

The purpose of the present review is to integrate these systems through a common sequence rather than organize the field as a catalogue of nanoparticles: formation/processing → structure → property → function → route-specific evidence → translational consequence. Five linked questions are used throughout. What processing step generates the relevant nanoscale architecture? Which physical or chemical property creates the claimed diagnostic or delivery function? How is that function altered by biological interfaces and administration route? Which experiment distinguishes a causal function from a decorative feature or surrogate endpoint? Finally, can the resulting product be defined and manufactured with sufficient reproducibility to support translation? This perspective deliberately emphasizes the convergence of diagnostics and delivery rather than any single host–guest chemistry or carrier family.

**Figure 2:**
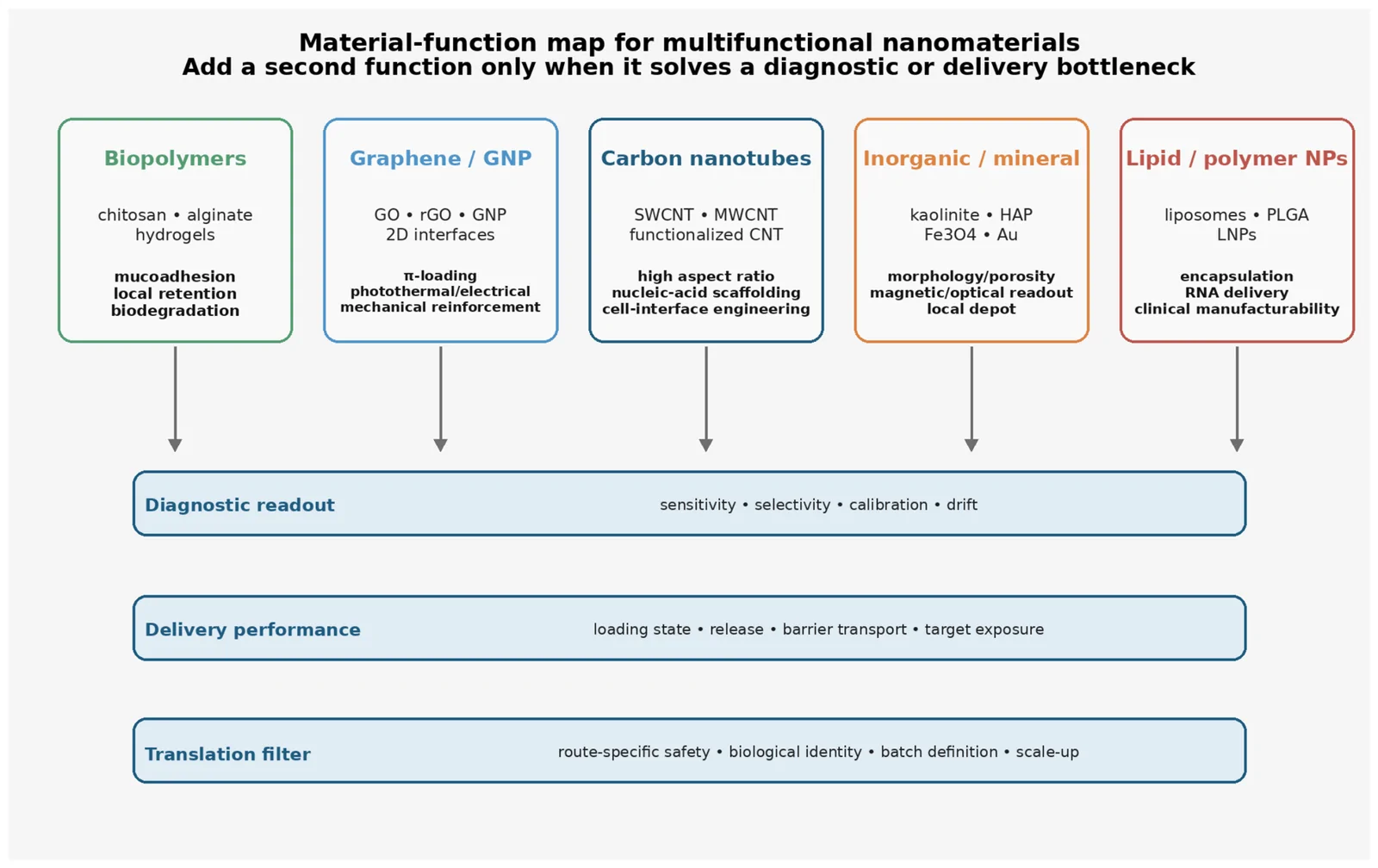
Material–function map for multifunctional nanomaterials. Different material classes offer distinct combinations of sensing, payload association, transport, triggering, and mechanical functions. The translational value of a second function depends on whether it improves diagnostic readout, delivery performance, or a defined product constraint. Original schematic prepared for this review.

The literature discussed here combines representative foundational studies with selected work available through August 2026. This is a narrative, design-oriented review rather than a systematic meta-analysis. Literature selection was organized around the title themes (AI-assisted sensing, barrier-directed transport, stimuli-responsive release, and theranostic integration) and the cross-cutting questions of formation/processing, geometry, biological identity, safety, characterization, and CQAs. Preference was given to independent studies that provide mechanistic, comparative, route-specific, negative/cautionary, or translational evidence; author-associated studies are retained only where they serve as specific case studies and are balanced with independent benchmarks. The framework is summarized in Figure 2, and the key design variables are listed in Table 1.

## 2. Nanomaterial-Enabled Diagnostics and AI-Assisted Biosensing

### 2.1. From Nanoscale Field Enhancement to a Diagnostic Decision

Nanomaterial-enabled biosensing is a natural entry point for precision nanomedicine because it exposes the difference between a physical signal and a useful diagnostic output. Metallic nanostructures, graphene, MXenes, diamond-related materials, and multilayer metamaterials can create strongly confined electromagnetic fields whose resonance depends on local refractive index, charge state, adsorption, or molecular composition. In surface plasmon, SERS, electrochemical, and terahertz devices, the primary layer of evidence remains conventional sensor performance: calibration, sensitivity, selectivity, limit of detection, reproducibility, drift, stability, interference testing, and behavior in realistic matrices [39]. Machine learning is an additional inference layer, not a substitute for this physical and analytical validation.

The gradient boosting-assisted sensor reported by Wekalao and co-workers is useful because the computational layer is coupled directly to the optical design. The proposed graphene-containing terahertz architecture was developed for simultaneous glucose and hemoglobin detection [5]. The numerical model produced distinct resonance responses and sensitivities of approximately 1000 GHz/RIU for glucose and 433 GHz/RIU for hemoglobin, with a reported figure of merit up to 23.81 RIU−1 and a quality factor of 22.7; a gradient-boosting regressor predicted modeled sensor behavior with approximately 93% accuracy. These values should be interpreted as design-space proof of concept, not clinical diagnostic performance. The unresolved steps are fabrication, experimental calibration, biological matrix testing, device-to-device variation, independent samples, and prospective diagnostic validation.

This distinction matters for the rapidly expanding literature on machine learning-assisted biosensors. An algorithm can interpolate a dense simulation space very accurately while failing with regard to fabrication variation, sensor drift, sample matrix effects, batch changes, or an unseen patient population. Accordingly, an AI-enabled sensor should report separate training, validation, and external test performance; the exact features presented to the model; uncertainty or calibration of the prediction; protection against information leakage; and the extent to which the dataset spans real device and biological variability [39]. A model trained only on numerically generated resonant frequencies demonstrates design-space learning, not clinical diagnosis. External validation is therefore essential for claims of generalizability, and prospective testing is required before an algorithm can be considered a reliable component of a real diagnostic decision.

### 2.2. Multiplexing, Polarization Independence, and the Value of Robust Readout

Multiplexed diagnostics impose a second constraint: two analytes should produce separable observables without requiring an impractical measurement geometry. Polarization-independent or weakly polarization-dependent structures can simplify instrument alignment and reduce one source of variance. At the same time, multiplexing increases the risk of spectral overlap, coupled resonances, and model overfitting. The most useful architectures create physically interpretable channels, for example two resonances with different analyte sensitivities, and then use machine learning to correct nonlinearities or combine several descriptors rather than asking the model to discover the entire sensing mechanism from opaque raw data.

Independent experimental studies clarify what the computational layer can add. Oiticica and co-workers applied computer vision and machine learning to microscopy images of plasmonic immunosensors and distinguished SARS-CoV-2 at 1 PFU/mL, whereas LSPR readout from the same immunosensor had a reported limit of detection of 10^3^ PFU/mL [6]. In a different setting, Heo and co-workers combined a plasmonic SERS platform with machine learning classification of osteoarthritis and rheumatoid arthritis patient samples [8]. A large 2025 case–control study further combined serum SERS with deep learning for multi-cancer early detection [7]. These examples move progressively from simulated response to experimental sensors and patient-derived data, but they also make the validation hierarchy clearer: impressive internal accuracy is not equivalent to performance across independent sites, instruments, batches, or prospectively recruited patients.

The future connection to drug delivery is not merely conceptual. A diagnostic material can define when, where, or how much therapy should be released. pH, glucose, inflammatory mediators, proteases, redox state, and tumor-associated molecular signatures are all candidate inputs to responsive delivery systems. The stronger design is therefore a closed loop in which the sensing variable is causally connected to the therapeutic decision. This is already emerging in integrated photonic probes that identify abnormal tissue chemistry, deliver or trigger therapy, monitor temperature or drug release, and reassess the local microenvironment after treatment [40,41,42]. The nanomaterial is most valuable when its diagnostic function reduces uncertainty in the intervention rather than simply providing an additional image.

### 2.3. What AI Adds Beyond Conventional Sensing—And What It Does Not

A conventional sensor converts a physical or chemical interaction into a calibrated observable. Machine learning is useful when the observable is multivariate, nonlinear, weakly separated, or affected by several nuisance variables. It can combine spectral bands, texture descriptors, multiple resonances, electrochemical features, or temporal trajectories; compensate nonlinear calibration; classify overlapping signatures; and quantify uncertainty when the training data support those operations [6,7,8,39]. The meaningful comparator is therefore not “AI versus no AI” in the abstract, but whether the same sensor, evaluated on the same independent samples, yields better discrimination, calibration, robustness, or decision utility when the model is added.

Conversely, machine learning cannot create molecular specificity that is absent from the sensing chemistry, rescue a poorly controlled fabrication process, or prove that a simulated resonance will survive biological fouling. Nor should model complexity be used to obscure a mechanistic sensor failure. Acceptable validation depends on intended use: simulation-space models require experimental fabrication and measurement; analytical classifiers require independent devices and realistic matrices; and clinically intended classifiers require external cohorts and prospective testing. The same logic is applied later to AI-driven formulation optimization and prediction of in vivo fate.

### 2.4. Formation → Structure → Property → Function as a Cross-Platform Chain

A function-first review must still begin with formation, because a material property is not independent of how the structure is made. For graphene-family systems, exfoliation, oxidation/reduction, lateral size selection, and covalent or non-covalent functionalization determine layer number, defect density, oxygen chemistry, accessible aromatic area, and aggregation. Those structural variables then control charge transport, optical response, adsorption, colloidal behavior, and ultimately sensing, loading, release, and biological interaction. For CNTs, growth method, purification, cutting/oxidation, and coating determine length distribution, residual catalyst, defect density, surface chemistry, and dispersion; the same variables govern payload association, cellular interaction, and toxicological response [32,33].

Lipid nanoparticles provide a complementary soft matter example. Rapid mixing, component ratios, ionizable lipid chemistry, PEG–lipid content, and cargo-to-lipid ratio generate an internal organization and surface composition that determine colloidal stability, nucleic acid protection, apparent pKa, protein interactions, endosomal escape, and tissue-level expression [3,4,20]. Reformulation studies demonstrate that changing component composition can shift organ accumulation and productive mRNA translation [16], while acid-degradable lipids show how a designed chemical transition can increase endosomal release and reduce persistence [17]. Thus, “formation → structure → property → function” is not an alternative to the function-first framework; it is the mechanistic path by which the function is created.

## 3. Mucosal and Local Interfaces: Biopolymer Nanoparticles and Hydrogels

### 3.1. Chitosan as an Interface-Active Nanocarrier

Chitosan occupies an important position in drug delivery design because its contribution is not limited to encapsulation. The polymer carries protonatable amino groups, can be processed into nanoparticles and gels, and can interact with negatively charged mucus, cell surfaces, and biological macromolecules. These properties support mucoadhesion, transient permeability enhancement, antimicrobial activity, and prolonged local residence, while source, molecular weight, degree of deacetylation, and processing can change performance. Recent independent and author-associated reviews both emphasize the breadth of chitosan formulations across small molecule, protein, nucleic acid, mucosal, wound, and theranostic applications, together with formulation-specific questions of immune response, hemocompatibility, and toxicity [9,10].

For multifunctional systems, chitosan is especially useful as an interfacial layer. A nanoparticle core can provide optical, magnetic, or high-area loading properties, while chitosan controls dispersion and biological contact. This division of labor is preferable to assuming that every component must directly bind the drug. Earlier chitosan-functionalized graphene oxide systems demonstrated that a biopolymer coating can improve aqueous handling while allowing drug and gene association with the underlying carbon surface [43]. In such hybrids, the chemically meaningful variables include degree of deacetylation, molecular weight, grafting or adsorption density, residual crosslinker, particle charge as a function of pH, and whether the coating masks or exposes the second material phase.

A common optimization error is to maximize positive zeta potential because it improves cell association in simple culture. For mucosal delivery, an excessively cationic surface can become immobilized in mucus rather than penetrating it; for systemic exposure, high charge can increase protein adsorption and rapid clearance. A more useful endpoint is productive transport: retention long enough to prevent immediate clearance, but not so strong that the particle is trapped before reaching its target. The same principle applies to wound delivery, where adsorption to the wound bed, exudate uptake, antimicrobial function, and release of a therapeutic payload need to be balanced against inflammation and tissue compatibility.

### 3.2. Alginate, Hydrogel Depots, and Chemical Stabilization of Labile Payloads

**Figure 3:**
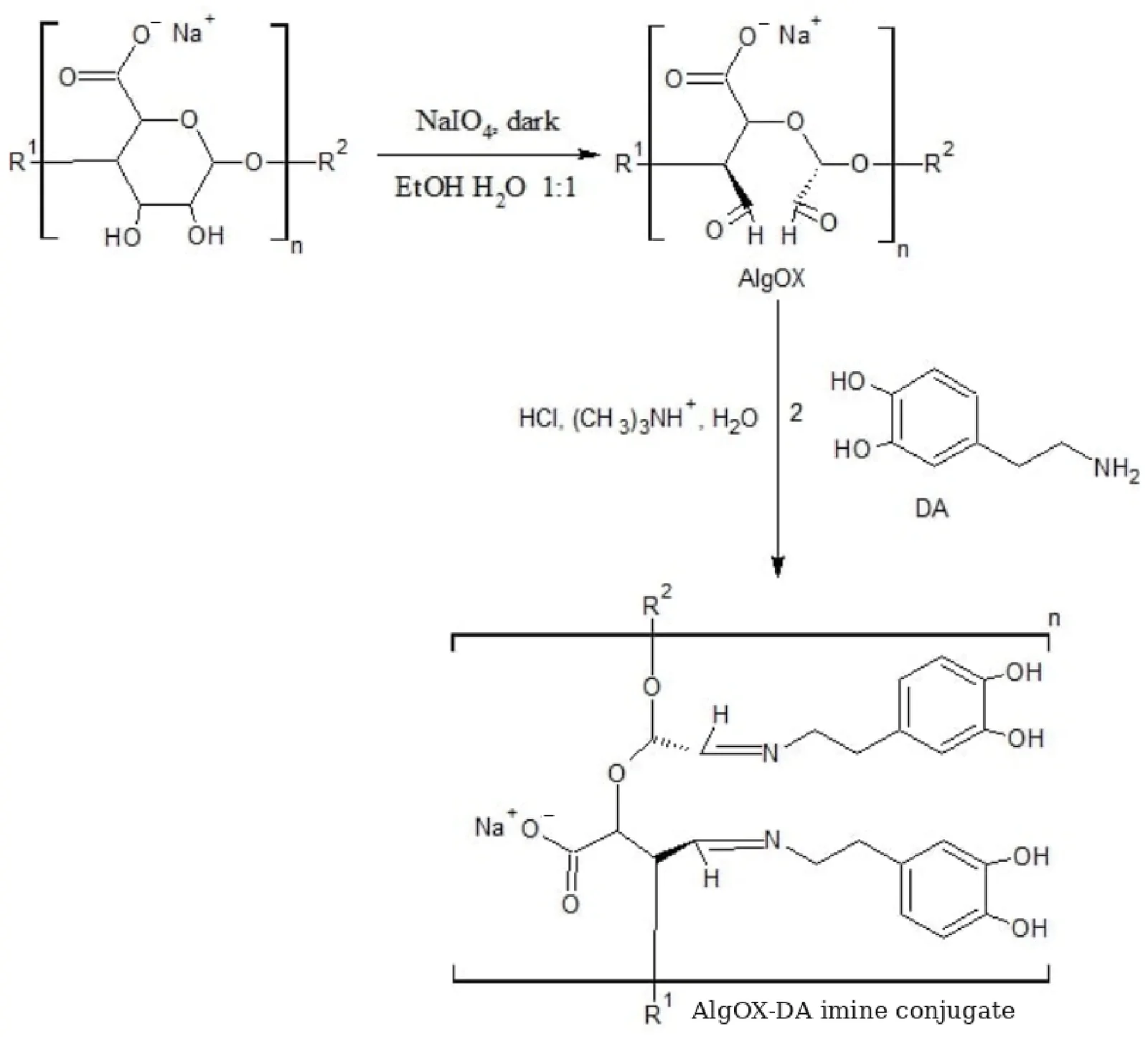
Synthetic pathway used to prepare the oxidized-alginate–dopamine (AlgOX–DA) imine conjugate. Reproduced and cropped from Trapani et al. [13], Materials 2021, 14, 3495, under the Creative Commons Attribution (CC BY 4.0) license.

Alginate provides a complementary design space. Its anionic polysaccharide backbone is readily gelled or oxidized, can be chemically conjugated, and can form soft matrices suitable for local delivery. Oxidation introduces aldehyde groups that permit Schiff-base chemistry with amines, enabling direct conjugation of molecules such as dopamine. Trapani and co-workers used this approach to prepare an oxidized-alginate–dopamine conjugate for nose-to-brain applications [13]. The synthetic route is reproduced in Figure 3 because it illustrates a general strategy: the polymer is not simply a passive matrix, but a chemically addressable carrier that can change payload stability, local retention, and release behavior.

**Figure 4:**
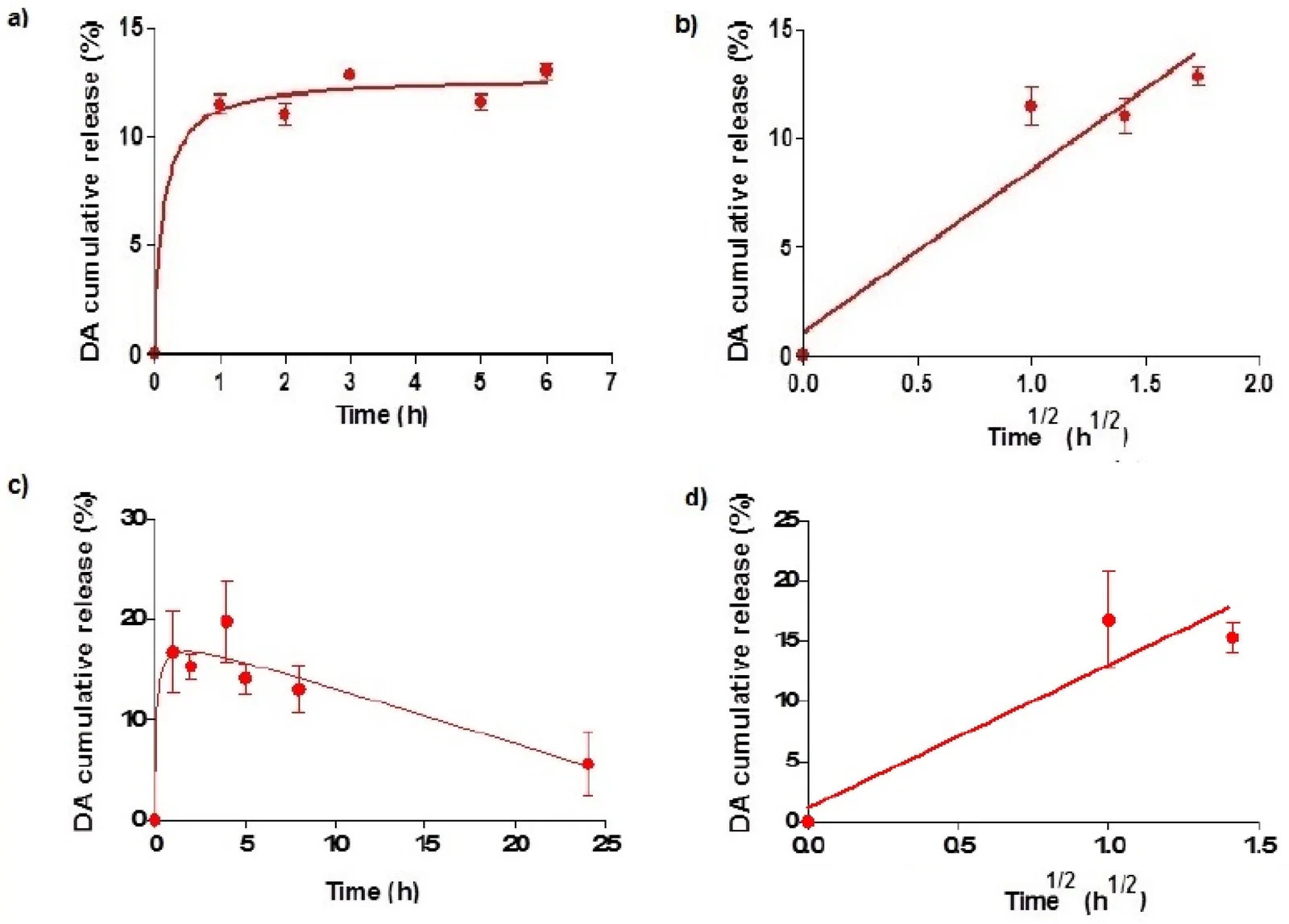
Dopamine release profiles from the AlgOX–DA imine conjugate: (**a**) DA cumulative release in simulated nasal fluid (SNF); (**b**) square-root-of-time dependence of DA released in SNF; (**c**) DA cumulative release in PBS (pH = 7.4); (**d**) square-root-of-time dependence of DA released in PBS (pH = 7.4). Reproduced and cropped from Trapani et al. [13], Materials 2021, 14, 3495, under the Creative Commons Attribution (CC BY 4.0) license.

The same study evaluated mucoadhesive behavior and dopamine release in simulated nasal electrolyte solution and phosphate-buffered saline. The release profiles in Figure 4 are particularly useful because they show how a chemically bound neuroactive payload can behave differently across media rather than following a universal “sustained release” curve. Medium composition and pH affect hydrolysis, oxidation, and the apparent release process. These data reinforce a broader rule for local delivery: stability of the released chemical species should be measured together with mass transfer. A carrier that releases a degraded drug at a controlled rate has not solved the delivery problem.

The later cell/particle work on oxidized-alginate–dopamine systems extended this chemistry toward biological interaction studies [14]. Together, these studies illustrate a useful progression from synthesis to physicochemical characterization, to route-relevant release, and then to cell interaction. That sequence is more translationally informative than adding several targeting groups before establishing whether the base material remains stable, reproducible, and biologically acceptable.

For local depots, “controlled release” should be decomposed into at least four measurements: total payload initially associated with the formulation, the fraction accessible for release, the chemical identity and activity of the released species, and the mechanism by which mass leaves the matrix. Hybrid nanoparticle–hydrogel systems can improve local retention while nanoparticles regulate release, but a slow curve caused only by bulk matrix erosion or drug degradation is not equivalent to stimulus-gated or diffusion-controlled delivery [11]. Release studies should therefore include mass balance, free drug controls, stability of the recovered payload, relevant sink conditions, and—where a stimulus is claimed—matched triggered and untriggered conditions. This distinction is used throughout the graphene, lipid, and inorganic sections below.

## 4. Nose-to-Brain Delivery: Designing for a Route Rather than a Particle

**Figure 5:**
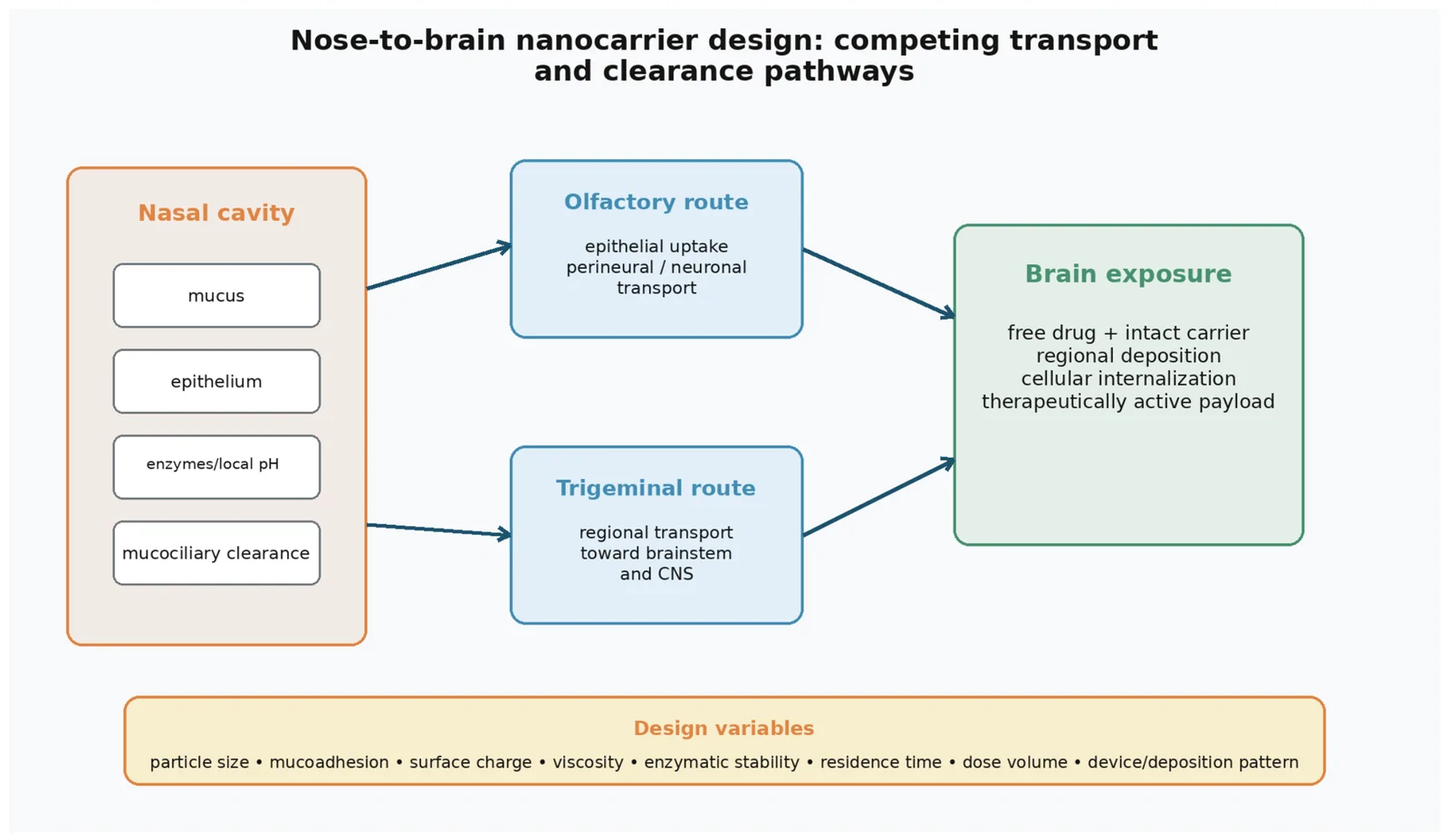
Nose-to-brain nanocarrier design map. Nasal deposition, mucus interaction, epithelial transport, enzymatic environment, mucociliary clearance, and olfactory/trigeminal pathways jointly determine brain exposure. Original schematic prepared for this review.

Intranasal administration is one of the clearest examples of why nanocarrier design should begin with anatomy and physiology rather than material novelty. The nasal route can provide non-invasive access to the central nervous system while reducing reliance on systemic circulation, but it confronts rapid mucociliary clearance, limited dose volume, enzymatic activity, heterogeneous deposition, epithelial barriers, and the need to reach olfactory or trigeminal pathways [15]. A particle with excellent serum stability may therefore be poorly suited to the nose, whereas a moderately adhesive or appropriately mucus-penetrating system can be advantageous if it increases residence without preventing transport [12,13,44]. Figure 5 maps these competing processes.

Sorrentino and co-workers developed optimized biopolymer-based nanoparticles for intranasal treatment of depressive disease, explicitly treating the blood–brain barrier as a route selection problem rather than only a nanoparticle-targeting problem [12]. The significance of this strategy is not that intranasal nanoparticles automatically “bypass” the BBB. Rather, formulation variables can improve deposition, protect a payload, prolong nasal contact, and alter the balance among local absorption, systemic uptake, and direct neural transport. Demonstrating brain delivery therefore requires comparative pharmacokinetics or biodistribution rather than inference from particle size alone.

The oxidized-alginate–dopamine platform adds a second design logic: conjugation can protect or retain a chemically unstable neurotransmitter while creating mucoadhesive behavior [13,14]. More broadly, the route cannot be validated by particle size, nasal administration, or a fluorescent brain signal alone. Shape and aggregation influence droplet behavior; mucus interaction influences residence; formulation and device geometry influence regional deposition; and labels may dissociate from the carrier. Direct-versus-systemic contribution, regional brain exposure, intact carrier fate, and pharmacologically active payload should therefore be distinguished wherever feasible [2,15,45].

External studies reinforce this route-specific interpretation. Cationic liposomes have been used for intranasal mRNA delivery and transfection in the brain [46]. Polyfunctional gold–iron oxide nanoparticles loaded with therapeutic microRNAs have combined intranasal delivery, imaging, and glioblastoma-directed therapy [47]. Anti-EphA3-functionalized gold nanoparticles conjugated with temozolomide provide another intranasal glioblastoma example [48]. In 2025, venlafaxine-loaded PLGA nanoparticles were reported to produce a rapid antidepressant effect in an animal model using a nose-to-brain strategy [44]. These systems differ substantially in material composition, but the decisive evidence is route-specific: nasal tolerability, deposition, residence, brain/plasma pharmacokinetics, regional distribution, carrier-versus-payload tracking, and comparison with free drug or systemic delivery. Representative systems spanning these approaches are compared in Table 2, while Table 3 makes the same evidentiary standard explicit for intranasal, intravenous, local depot, wound, and tumor-interventional routes.

## 5. Interface-Regulated Loading and Stimuli-Responsive Release: Graphene and Hybrid Hydrogels

### 5.1. Carbon Interfaces as Loading Surfaces and Functional Fillers

Graphene-family materials remain attractive for drug delivery because a single material can provide a large aromatic surface, mechanical reinforcement, electrical conductivity, and optical or photothermal response. Aromatic and hydrophobic drugs can associate with sp2-rich surfaces through π interactions and hydrophobic forces, while oxidation or polymer coatings provide sites for covalent attachment and improve aqueous dispersibility [24,25]. The relevant structure is created by exfoliation, oxidation/reduction, lateral size selection, and functionalization; these steps determine accessible aromatic area, defect density, surface oxygen chemistry, and aggregation. The same variables control drug affinity, release, optical response, biological identity, and toxicity. A useful graphene carrier must therefore be defined as a material population and processing history, not merely by the word “graphene”.

Early PEGylated nanographene oxide work established that surface modification could support delivery of poorly water-soluble anticancer agents [24], and nano-graphene oxide was subsequently developed for cellular imaging and drug delivery [25]. Chitosan-functionalized graphene oxide extended this concept to hybrid drug and gene carriers [43]. Stimuli-responsive variants exploited acidic conditions, redox-cleavable coatings, ATP-responsive assemblies, photothermal effects, and magnetic components [26,27,28,29,30,31]. These studies remain mechanistically useful because they reveal several distinct release mechanisms: weakening of drug–surface interactions after protonation, cleavage of a surface tether, structural disassembly, local heating, or magnetic guidance. They should not be grouped under a generic label of “smart release” without identifying the actual material transition.

### 5.2. Graphene as a Hydrogel Modifier

Hydrogels provide a particularly informative setting for graphene because the carbon phase can alter a network that already has its own diffusion, swelling, and degradation behavior. Mauri and co-workers demonstrated graphene-laden hydrogels as thermally triggered delivery systems [21]. More recent work investigated few-layer graphene incorporated into a polyacrylic acid/agarose hydrogel for dopamine delivery [49]. The graphene was intended to create additional interactions with dopamine, improve dispersion within the polymer matrix, and influence release while the hydrogel provided the hydrated depot. Dopamine remained detectable after 24 h of release, supporting the idea that the combined matrix can slow degradation of a labile molecule [49].

The important design lesson is that graphene need not be the primary carrier. In a composite depot, it can function as a nanoscale regulator of local binding, heat transfer, conductivity, or mechanical stability. This reduces the amount of nanocarbon required and can favor local rather than systemic administration, potentially simplifying biodistribution questions. The same principle appears in sodium alginate/graphene nanoplatelet systems for resveratrol, where the hybrid structure was used to control loading and release; at pH 6.8, the alginate–resveratrol system released about 54% of the loaded drug, and incorporation of GNPs altered the material and release behavior [50]. The most useful comparison is therefore not “graphene versus no graphene” in isolation, but whether the graphene-containing composite provides a measurable kinetic or stability advantage without unacceptable changes in biocompatibility.

### 5.3. Triggered Release Requires a Trigger Budget

**Figure 6:**
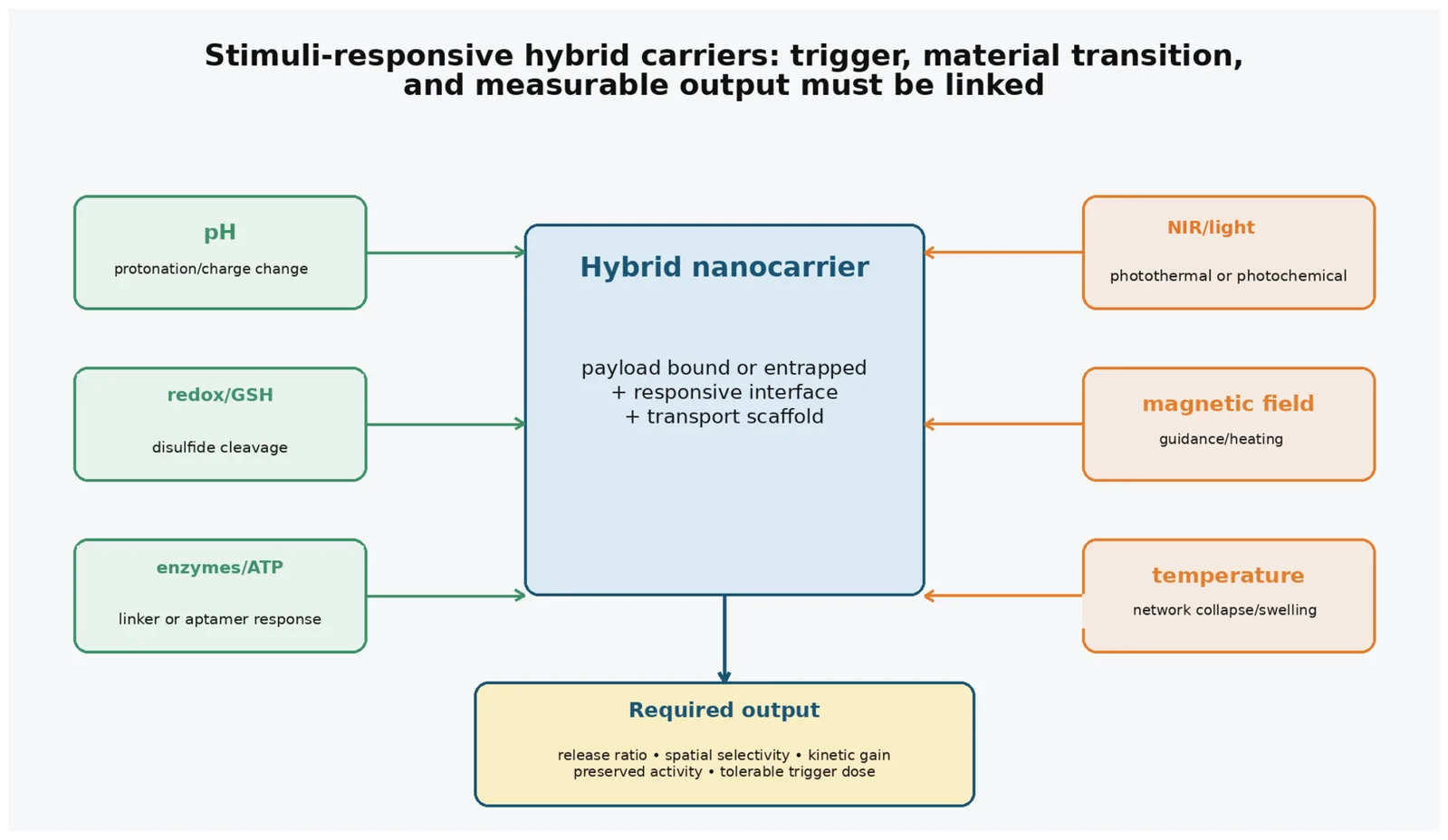
Stimuli-responsive hybrid carriers. Endogenous and exogenous triggers are useful only when a defined material transition produces a measurable and biologically relevant output at a tolerable trigger dose. Original schematic prepared for this review.

Stimuli-responsive carriers are often presented as binary switches, but biological triggers have finite amplitude, and external triggers have a finite safety budget. Tumor acidity may differ from surrounding tissue by less than the pH change used in a proof-of-concept buffer experiment. Intracellular glutathione can cleave disulfides, but access depends on uptake and endosomal escape. Near-infrared irradiation can generate heat, but the temperature distribution and optical penetration are tissue-dependent. Magnetic actuation depends on field strength, particle magnetic moment, and spatial gradients. A robust study should therefore report the trigger threshold, the baseline leak in the absence of trigger, the fold change after activation, the reversibility or irreversibility of the transition, and whether the trigger itself changes cell viability. The relationship among trigger, material transition, and measurable output is summarized in Figure 6.

A second interpretive problem is to distinguish genuine trigger-controlled release from accelerated matrix breakdown. Evidence for responsiveness should show that the stimulus causes a defined material transition and a reproducible change in release rate or released dose relative to an otherwise matched untriggered control. The released molecule should remain chemically and pharmacologically intact, and a mass balance should distinguish payload release from matrix fragments. If the trigger simply destroys the carrier or produces nonspecific leakage at a damaging dose, the system is stimulus-sensitive but not necessarily a useful controlled release platform.

This “trigger budget” also helps compare nanomaterials with devices. A fiber optic interventional system can deliver heat exactly where a thermoresponsive formulation is located, thereby avoiding the need for whole-body distribution of a photothermal nanomaterial [41,42]. Conversely, a systemically delivered photothermal nanoparticle may be appropriate when distributed tumor deposits must be treated. The choice is therefore not simply between materials; it is between intervention architectures with different exposure, control, and safety profiles.

## 6. High Aspect Ratio Nanocarriers: Carbon Nanotube Formation, Delivery, and Safety

### 6.1. Functionalization Converts a High Aspect Ratio Material into a Biological Carrier

Carbon nanotubes provide an unusually high surface-to-volume ratio, extended aromatic walls, and a geometry that can interact with cell membranes. These properties created early interest in drug, protein, and nucleic acid transport but also raised concerns about persistence, metal catalyst residues, fiber-like behavior, and aggregation [32,33]. Their final biological identity is generated by the full processing chain: synthesis fixes wall number and an initial length/catalyst distribution; purification changes residual metals and carbonaceous impurities; cutting or oxidation changes aspect ratio, defects, and surface oxygen; and coating or covalent functionalization changes colloidal stability, charge, payload association, and protein adsorption. Functionalization is therefore not an accessory step—it produces a new material whose delivery and safety must be re-characterized.

**Figure 7:**
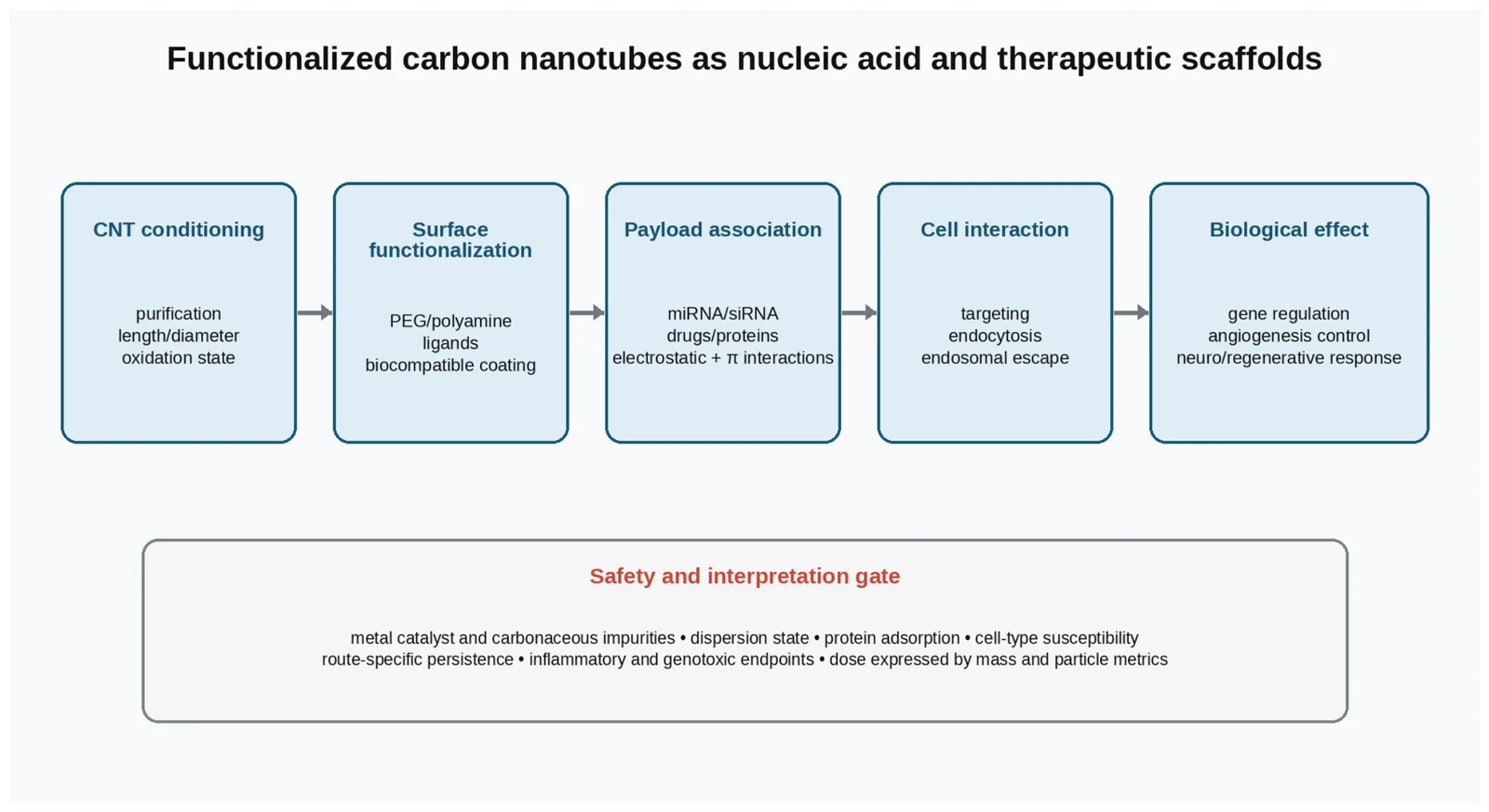
Functionalized carbon nanotubes as therapeutic and nucleic acid scaffolds. Purification and surface chemistry determine payload association, cell interaction, biological effect, and safety. Original schematic prepared for this review.

Masotti and co-workers used polyamine-coated carbon nanotubes to deliver microRNAs into endothelial cells and regulate angiogenesis [22]. This is a qualitatively different application from adsorption of a small hydrophobic drug. The carrier must compact or associate with the nucleic acid, protect it, support cellular uptake, allow intracellular release, and preserve the biological activity of the RNA. The endpoint is consequently gene regulation and a cellular phenotype, not only loading efficiency. Figure 7 summarizes this sequence and the associated safety gate.

CNTs have also been used as targeted immunological scaffolds. PEG-modified single-walled nanotubes armed with antibodies against GITR showed selective targeting of intratumoral regulatory T cells in earlier work [23], illustrating how high aspect ratio carriers can display biologically active ligands. Such targeting claims, however, depend on the disease-specific receptor landscape and should be evaluated against uptake by the liver, spleen, macrophages, and other non-target compartments. The larger nanomedicine literature has repeatedly shown that target binding does not guarantee efficient tissue delivery [1].

For delivery, the strongest endpoint depends on payload. Small molecule adsorption should be followed by release and preserved drug activity; nucleic acid binding should be followed by protection, intracellular release, and target gene regulation. Increased cell association is only an intermediate step. Device compatibility is also route-specific: aerosolization and droplet stability matter for inhaled or nasal concepts, whereas serum stability, protein adsorption, circulation, and phagocyte uptake matter for intravenous exposure. Treating these variables as a chain prevents “high loading” or “high uptake” from being used as surrogates for successful delivery.

### 6.2. Toxicology Is a Material Property Map, Not a Single CNT Label

CNT toxicology cannot be summarized by one threshold concentration. Diameter, length, wall number, surface oxidation, residual catalyst, adsorbed dispersant, agglomeration, dose metric, exposure route, and cell model all matter [32,33]. Comparative studies across carbon nanomaterials have likewise shown that dispersion state and functionalization change the effective biological dose [51,52,53]. A formulation that is more dispersible may expose cells to more individual nanotubes even when the nominal mass concentration is unchanged.

Purity is especially important for carbon nanomaterials. Transition metal catalysts and carbonaceous by-products can confound toxicity measurements, while aggressive oxidative purification introduces defects and oxygen-containing groups. The appropriate safety strategy is therefore to characterize the material before and after functionalization, quantify relevant impurities, report dispersion state under the exposure conditions, and relate biological effects to both mass and particle/geometry metrics. This is more informative than attributing an effect generically to “CNTs”.

## 7. Nucleic Acid Packaging and Endosomal Escape: Lipid Nanoparticles and Soft Nanostructures

### 7.1. Formation and Internal Structure Are Functional Variables

Lipid nanoparticles (LNPs) correct an imbalance in the original literature coverage because they provide both a mechanistically rich material system and the strongest clinical precedent for nanoscale nucleic acid delivery. Contemporary LNPs are not simple spherical containers. Ionizable lipid, helper phospholipid, cholesterol or alternative structural lipids, PEG–lipid, nucleic acid, mixing conditions, and formulation pH jointly determine particle formation and internal organization [3,4]. Surface-sensitive NMR studies show that even the distribution and conformation of surface PEG and ionizable lipid can be resolved in intact mRNA LNPs, linking composition to colloidal and biological behavior [20].

The function chain is therefore explicit: formulation and mixing create an internal nanostructure and surface composition; these determine cargo protection, apparent pKa, serum interactions, cellular uptake, endosomal destabilization, and finally cytosolic nucleic acid availability. Total mRNA associated with an LNP is not the decisive endpoint. Productive delivery requires that the cargo remain intact during transport, reach the relevant cells, escape the endosome, and produce the intended protein or gene regulatory effect. This distinction mirrors the earlier rule that high loading is not equivalent to bioavailable payload.

### 7.2. Endosomal Escape, Organ Targeting, and Route-Specific Evidence

Endosomal escape is a major rate-limiting step for nucleic acid carriers. Reviews of LNP design therefore distinguish circulation and organ accumulation from intracellular release [4]. Acid-degradable LNPs provide a mechanistically informative example: an acid-labile linker was engineered to hydrolyze rapidly in endosomes, producing a defined material transition associated with improved mRNA delivery in multiple tissues [17]. This is stronger evidence of responsiveness than observing cargo uptake without demonstrating cytosolic function.

Organ targeting likewise requires a productive endpoint. Reformulation of LNP composition has been shown to shift both organ accumulation and mRNA translation, emphasizing that fluorescence or particle signal alone does not establish useful delivery [16]. Engineered BBB-crossing lipids have enabled systemic mRNA delivery and expression in neurons and astrocytes in preclinical models [19]. These studies should be read with the same route-specific standard used for nose-to-brain systems: accumulation, intact carrier or cargo tracking, cellular localization, productive expression, toxicity, dose schedule, and comparator formulations must be separated. The translational advantage of LNPs does not remove the need to define repeated-dose immunological effects, complement interactions, persistence, and manufacturing CQAs.

### 7.3. Geometry Beyond Spheres: Vesicles, Bilayers, and Lipid Nanotubes

Geometry is not unique to CNTs or rigid inorganic particles. Soft lipid assemblies can adopt vesicular, tubular, lamellar, or other morphologies depending on molecular packing, composition, curvature stress, and processing. Villanueva and co-workers provided a useful formation–structure example in which bilayer composition controlled nanomechanical behavior and the spontaneous formation of helical lipid nanotubes [54]. The lesson for drug delivery is broader than that specific system: composition can determine nanoscale architecture, and architecture can change accessible surface, encapsulation volume, membrane mechanics, interaction with cells, and release pathways. Geometry should therefore be treated as a CQA when it is mechanistically linked to function, not as a descriptive microscopy result.

## 8. Geometry, Porosity, Magnetic, and Plasmonic Platforms

### 8.1. Morphology and Pore Architecture as Drug Delivery Variables

Inorganic carriers contribute properties that soft polymers do not easily provide: rigid pore architecture, ion exchange, defined crystallographic surfaces, magnetism, high atomic number, and localized plasmon resonances. Their principal translational challenge is equally distinctive: the inorganic component may persist, dissolve into biologically active ions, accumulate in phagocytic organs, or create long-term inflammatory effects. For this reason, inorganic nanomaterials are particularly compelling when geometry, porosity, imaging, or actuation supplies an indispensable function and exposure can be adequately controlled.

The kaolinite study by Alqahtani and co-workers provides a clear demonstration of morphology-driven loading [34]. Transforming raw kaolinite into separated nanosheets or nanoscroll/nanotube morphologies increased the reported oxaliplatin loading capacity from 29.6 mg/g for the raw mineral to approximately 304.9 mg/g for nanosheets and 473 mg/g for nanotubular structures. The fitted active site density also increased substantially. This example is useful because the chemical composition of the mineral is not the only variable: exfoliation and scrolling modify accessible area, steric environment, and diffusion paths. A rational inorganic carrier therefore requires characterization of shape distributions and accessible surface, not only elemental composition.

Nanoporous systems make the geometry–function relation even more explicit. Mesoporous silica is a representative case because pore diameter, pore volume, accessible surface area, connectivity, surface chemistry, and diffusion path can determine the fraction of payload that can enter the structure and the rate at which it can leave [37]. Reporting nominal BET surface area or total loading alone is therefore insufficient; pore accessibility in the relevant medium, free versus pore-associated drug, desorption/release kinetics, and the chemical integrity of released payload should be resolved. For persistent inorganic carriers, degradation and clearance must be measured as separate design variables because the same pore architecture that favors loading can also alter dissolution and tissue persistence [38].

Surface functionalization provides a complementary inorganic example in which the added organic phase must change a measurable delivery variable rather than merely increase compositional complexity. A zinc phosphate/hydroxyapatite hybrid modified with chitosan or β-cyclodextrin showed that surface chemistry can alter hydration, accessible binding sites, 5-fluorouracil loading, and release behavior [35]. This specific case is consistent with independent hydroxyapatite literature showing that organic functionalization changes interfacial hydration, protein adsorption, colloidal behavior, and drug association [36]. The relevant comparison is therefore bare versus functionalized inorganic carrier under matched conditions, with loading, releasable fraction, chemical activity, and biological compatibility evaluated together.

### 8.2. Magnetic and Gold-Based Theranostics

Magnetic iron oxide and gold nanostructures are frequent theranostic building blocks because they can contribute imaging, magnetic guidance, photothermal conversion, or radiosensitization while also carrying drugs or nucleic acids. The intranasal gold–iron oxide microRNA system developed for glioblastoma combined therapeutic microRNAs with multimodal imaging and presensitization to temozolomide [47]. Anti-EphA3-functionalized gold nanoparticles carrying temozolomide provide a second brain tumor example in which ligand targeting and intranasal administration were combined [48]. These platforms are strongest when the diagnostic component changes treatment planning or confirms delivery, rather than merely decorating an otherwise conventional formulation.

The opposite outcome is equally informative. Dulf and co-workers examined doxorubicin incorporated into gold nanoparticles in vivo and found that nanoparticle incorporation did not necessarily eliminate cardiac toxicity [55]. Such negative or cautionary results are essential to the field because they show that targetability or nanoscale packaging does not guarantee a safer exposure profile. A formulation can change tissue distribution, oxidative stress, or drug release in ways that offset the intended benefit. Comparative toxicology against the free drug and carrier-only control should therefore be built into theranostic development from the beginning.

### 8.3. From Theranostic Particles to Closed-Loop Intervention

Recent work is moving beyond “image plus therapy” toward closed-loop intervention. A 2026 integrated theranostic nanoplatform combined radionuclide-based imaging, NIR-II fluorescence, targeting, and phototherapeutic functions to connect diagnosis, image-guided intervention, and treatment [40]. In parallel, a multifunctional fiber optic probe has been designed to identify tumor boundaries through pH sensing, perform photothermal therapy with simultaneous temperature monitoring, and reassess the local tumor microenvironment after treatment [41]. Another fiber optic platform couples localized heating with thermoresponsive doxorubicin-loaded microgels and real-time thermal monitoring [42]. These systems show that closed-loop precision treatment may be achieved by combining nanomaterials with devices rather than forcing every function into a systemically circulating nanoparticle.

This distinction has practical implications. Device-assisted local therapy can reduce the amount of persistent nanomaterial introduced into the body, provide direct spatial control, and simplify trigger dosimetry. Systemic nanotheranostics, however, can address disseminated disease and provide whole-body imaging. The appropriate architecture depends on disease distribution, accessibility, and the therapeutic window. The common requirement is measurable feedback: the diagnostic or sensing channel should verify location, dose, response, or failure in a way that changes the next action. Representative external benchmark systems spanning these intervention architectures are compared in Table 4.

## 9. Biological Identity, Biodistribution, and Route-Specific Safety

A nanomaterial enters a biological system with a synthetic identity but rapidly acquires a biological one. Proteins, lipids, metabolites, ions, and other biomolecules adsorb to particle surfaces and can alter aggregation, receptor accessibility, uptake, complement activation, and organ distribution. The resulting corona is dynamic and material-specific. Large interlaboratory efforts have shown that even protein corona measurement itself can vary with sample preparation and analytical pipeline [56,57], and LNP studies show that corona formation can change delivery outcomes and apparent tropism [58]. Broader analyses of nanomedicine biodistribution emphasize that circulation, tissue accessibility, cellular uptake, payload behavior, and clearance are distinct steps that should not be collapsed into a single “targeting” label [2].

For multifunctional materials this problem is amplified because the surface often carries the diagnostic or targeting function. A protein layer can shield a ligand, change the local refractive index of a plasmonic sensor, occupy a hydrophobic carbon surface, or alter magnetic particle uptake. In mucosal systems, mucus proteins and glycoproteins play an analogous role. The relevant biological medium should therefore be treated as part of the formulation. Size, zeta potential, release, optical response, and binding measured only in water or simple buffer are insufficient when the intended use involves serum, wound exudate, nasal mucus, or intracellular compartments.

Biodistribution should be interpreted in terms of carrier, payload, and analytical label. A high concentration of nanoparticle-associated fluorescence in an organ does not establish that intact carrier or pharmacologically active drug is present there, nor does a drug assay alone show whether intact particles remain. Dual-labelled nanoparticles have demonstrated that label dissociation can materially distort apparent in vivo fate [45]. Orthogonal or dual tracking is therefore preferred where feasible, especially for triggered systems in which release is supposed to occur only after target arrival. Without separate measurements, apparent “targeting” can reflect label redistribution, release in circulation followed by conventional drug distribution, or carrier uptake without productive payload delivery.

Carbon materials make these issues especially visible. Graphene-family toxicology varies with lateral dimension, oxidation state, functionalization, aggregation, dose, and route [51,52,53]. Functionalization can improve dispersion and reduce some forms of membrane damage, yet better dispersion may also increase the number of individual particles available for cellular interaction. CNTs add aspect ratio and catalyst impurities to this parameter space [32,33]. Inorganic carriers add dissolution and persistence, while cationic biopolymers add membrane and immune effects that depend on charge density. Safety claims therefore need to be derivative-, morphology-, and route-specific.

The practical implication is that toxicology should be embedded in the design matrix rather than deferred until after efficacy optimization. Route-specific risk categories should be explicit. Repeated intranasal exposure requires mucosal, ciliary, inflammatory, and local histopathological evaluation. Intravenous exposure requires hemocompatibility, coagulation, complement activation, phagocyte uptake, and organ accumulation; complement is itself a mechanistic contributor to nanoparticle recognition by blood phagocytes [59]. Persistent inorganic carriers require dissolution, degradation, long-term retention, and clearance data. For all routes, carrier fate should be compared with payload fate, and the exposure range used for efficacy should overlap the range used for safety. For photothermal systems, off-target thermal injury is part of material system safety even if the photothermal agent itself is chemically inert.

## 10. Machine Learning as a Cross-Cutting Tool for Nanomaterial Design

Machine learning can contribute at three distinct stages of nanomedicine development: (i) interpretation of sensor signals, (ii) optimization of formulation, composition, and process variables, and (iii) prediction of biological distribution or efficacy. These applications should not be conflated, because their target variables, sources of bias, and validation standards differ. In a biosensor, the model maps a measured response to analyte identity, concentration, or clinical class. In formulation development, it maps composition and processing variables to particle attributes or biological performance. In in vivo prediction, it maps a description of the nanoparticle and experimental context to tissue distribution or therapeutic outcome.

### 10.1. Sensor Interpretation

The Wekalao study illustrates design-space learning for a multiplexed graphene-based sensor [5], while experimental plasmonic and SERS studies show how models can classify sensor images, spectral fingerprints, or patient-derived biofluid measurements [6,7,8]. For this use case, acceptable validation progresses from internal cross-validation to independent devices and sample matrices, then to external patient cohorts, and finally prospective testing when the output is intended to guide a diagnostic decision. A model that never encounters independently acquired experimental data should not be described as clinically validated.

### 10.2. Formulation and Material Optimization

Formulation optimization can benefit from machine learning because composition spaces are high dimensional and experimentally expensive. Existing work combines data-driven and molecular/physics-based modeling to prioritize nanocarrier designs [60,61]. A particularly relevant independent example used AI models to screen ionizable-lipid structures for apparent pKa and mRNA delivery efficiency, followed by synthesis and mouse testing of model-selected candidates [18]. That prospective manufacture-and-test step is critical: it converts retrospective correlation into evidence that the model can nominate new materials with useful performance. Models that optimize only size or encapsulation efficiency should not be assumed to optimize target site exposure or therapeutic effect.

### 10.3. Prediction of In Vivo Fate

Prediction of in vivo fate is potentially transformative but especially vulnerable to hidden confounding. Mi and co-workers used machine learning models to predict tissue distribution and tumor delivery of nanoparticles in mice [62], showing that data-driven models can extract relationships between formulation descriptors and biodistribution. However, the target variable depends on animal model, disease state, dose, sampling time, administration route, analytical method, and whether carrier, label, payload, or biological activity is measured. A model trained across heterogeneous literature data can learn laboratory practices as easily as nanomaterial biology. External validation across laboratories and prospective testing of model-nominated candidates are therefore essential before such a model can guide in vivo decisions.

### 10.4. Validation Standards for AI-Enabled Nanomedicine

Data quality is a critical quality attribute for AI-enabled nanomedicine. Minimum requirements include explicit descriptor definitions, transparent preprocessing, independent test sets, reporting of missing data, prevention of information leakage, uncertainty or calibration estimates, and version control. External validation should be treated as essential rather than optional whenever generalization is claimed. Prospective validation should be required when a model is intended to choose a formulation, material, or clinical action: the model should make a locked prediction, the candidate should then be manufactured or the patient/sample prospectively evaluated, and performance should be compared with a predefined benchmark. Clinical models additionally require subgroup performance, calibration, clinically meaningful comparators, and evidence that model output changes a decision rather than merely reproducing an internal label.

A further opportunity lies in joining diagnostic and delivery data. A sensor may define a biological phenotype, a model may classify the state, and a responsive carrier or local device may apply an intervention appropriate to that state. The post-treatment signal can then provide a new label for model improvement. This is a genuine closed learning loop, but it also raises a regulatory challenge because both the material product and the computational model can change. Locked models, version control, predefined update rules, and traceable manufacturing data will be necessary if adaptive systems are to move beyond research prototypes.

## 11. Product Definition, Critical Quality Attributes, and Translation

The translation of multifunctional nanomaterials is often limited less by proof-of-concept efficacy than by product definition. A conventional small molecule can be specified primarily by chemical identity, purity, dose, and formulation. A nanomaterial product may require distributions of size, morphology, surface composition, ligand density, free and associated drug, residual solvents or catalysts, colloidal stability, and release behavior. A diagnostic nanomaterial adds calibration, signal drift, batch-to-batch optical or electrical response, and potentially model version. A responsive system adds trigger threshold and activation reproducibility. These variables can interact, which is why adding functions without a mechanistic need can make a promising prototype difficult to manufacture.

Advanced characterization should be question-driven and orthogonal. Dynamic light scattering provides hydrodynamic size but can obscure multimodal populations; electron microscopy resolves morphology but not necessarily the hydrated state; chromatography and field-flow fractionation can separate free cargo or particle subpopulations; scattering and spectroscopy can interrogate internal or surface structure. Graewert and co-workers coupled asymmetrical flow field-flow fractionation with small-angle X-ray scattering to obtain size-resolved information on mRNA nanoparticles [63], while surface-sensitive NMR has resolved lipid distributions in intact mRNA LNPs [20]. The appropriate combination depends on the claim. To distinguish total drug loading from accessible or releasable drug, the free fraction should be separated and quantified, release should be measured under relevant sink conditions, and recovered drug identity/activity should be verified. To establish whether an in vivo signal represents intact carrier rather than released cargo or label, dual or orthogonal tracking is needed [45]. A routine checklist of DLS plus microscopy is insufficient when the claimed function depends on payload state, pore accessibility, carrier integrity, or biological transformation.

Critical quality attributes should be selected from the mechanism of action and the route. For a nose-to-brain nanoparticle, aerosol or spray properties, colloidal stability after actuation, mucosal interaction, and payload stability may be more important than an extremely narrow size distribution in the original vial. For an LNP, lipid composition, apparent pKa, surface PEG state, cargo integrity, free cargo, particle distribution, potency, and stability are linked to productive nucleic acid delivery [3,20]. For a graphene hydrogel depot, filler distribution, drug–graphene interaction, swelling, mechanics, released-drug integrity, and trigger response are central. For a plasmonic biosensor, resonance reproducibility, surface functionalization, fabrication tolerance, nonspecific adsorption, calibration, and model version are CQAs. For a CNT carrier, purity, aspect ratio, surface chemistry, payload complexation/release, and inflammatory response are unavoidable.

**Figure 8:**
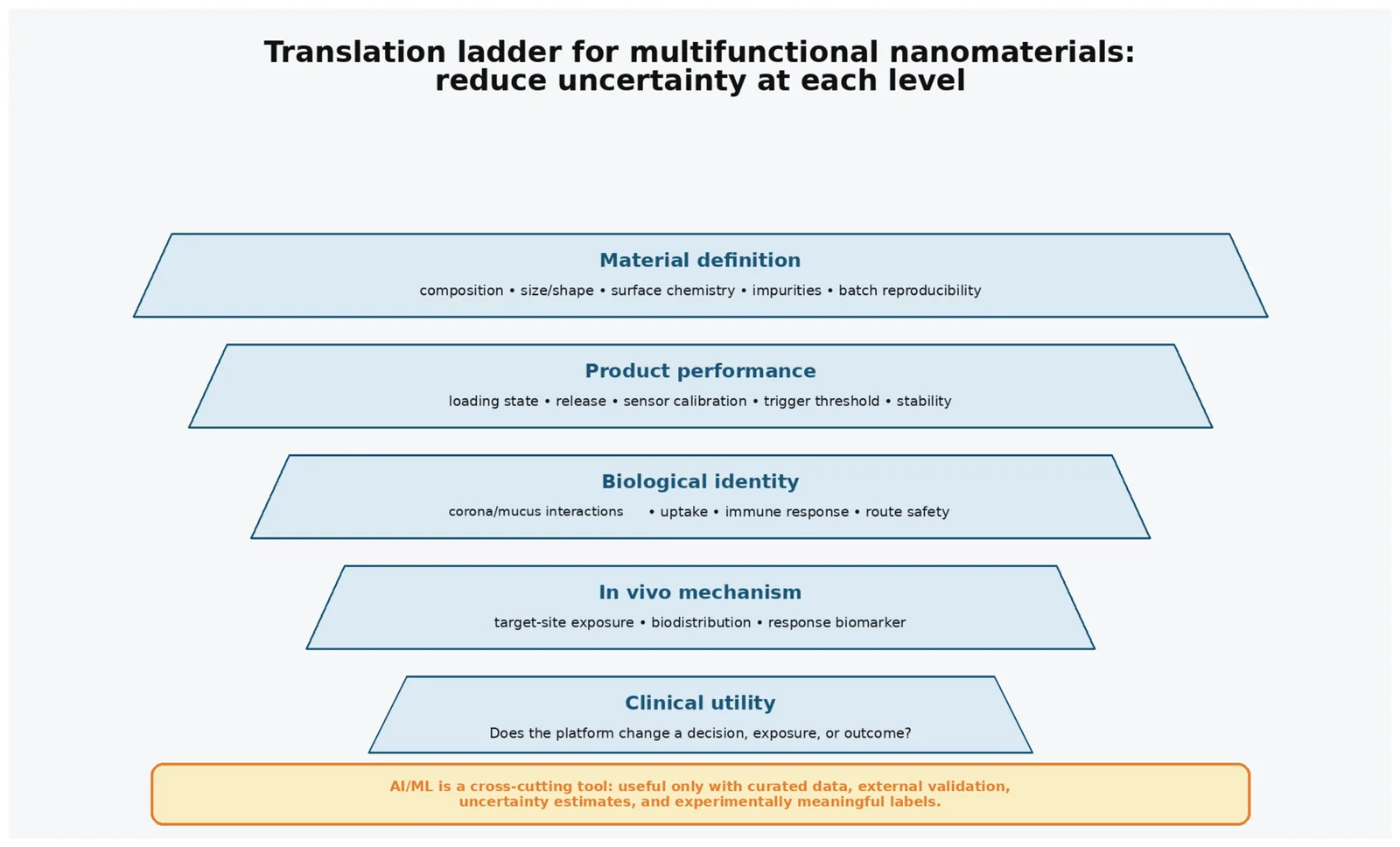
Translation ladder for multifunctional nanomaterials. Material definition, product performance, biological identity, in vivo mechanism, and clinical utility should be resolved progressively. AI/ML can support all levels but does not substitute for experimentally meaningful labels and external validation. Original schematic prepared for this review.

Manufacturing strategy should be considered early. Batchwise sonication, poorly controlled oxidative treatment, heterogeneous natural polymers, rapid-mixing conditions, and manual surface functionalization can all generate hidden variability. Scale-up changes mixing, heat transfer, shear, nucleation, self-assembly, and aggregation. Sterilization can alter polymer molecular weight, nanoparticle surface chemistry, or sensor coatings; lyophilization can improve storage yet change redispersion. The translational objective is not to preserve the laboratory process, but to preserve the product attributes that create the mechanism. Quality-by-design and process analytical technology are therefore natural partners for machine learning, provided that models are trained on measured process and product data. Figure 8 summarizes the translation ladder, while Table 5 elevates the CQA concept into a minimum-evidence roadmap from material definition to translational utility.

## 12. Outlook: From Multifunctionality to Verifiable Precision

The next stage of nanomedicine is unlikely to be defined by a single superior material class. Biopolymers, carbon nanostructures, lipid nanoparticles, nanoporous and magnetic materials, plasmonic surfaces, and hydrogels solve different problems. Their most productive use will be modular: a material is selected because it supplies a function that is difficult to obtain otherwise, and that function is validated against a relevant biological constraint. The same rule explains why a mucoadhesive polymer can be more valuable than a complex targeting ligand for some intranasal formulations, why graphene may be more credible as a local hydrogel modifier than as a persistent systemic carrier in some applications, why LNP chemistry must be judged by productive nucleic acid expression rather than accumulation alone, and why a fiber optic device can outperform a circulating particle when the lesion is physically accessible.

Diagnostics and delivery are also converging in a deeper sense. Nanomaterial sensors increasingly generate multidimensional signals that require computational interpretation, while delivery platforms increasingly need biomarkers to determine when and where an intervention should occur. The most compelling systems will connect these two domains through measurable feedback. An analyte or local physical state should trigger a decision; the intervention should change a defined mechanistic variable; and the response should be observed with sufficient temporal and spatial resolution to determine whether the intended mechanism occurred.

Artificial intelligence can accelerate this transition, but only if algorithmic complexity is not substituted for experimental causality. Sensor interpretation, formulation optimization, and in vivo prediction are distinct use cases and should carry distinct validation packages. A model that predicts resonance from geometry can speed device optimization; a model that selects an ionizable lipid can reduce formulation screening; a model that forecasts biodistribution can prioritize candidates. In each case, external validation is necessary for claims of generalization, and prospective testing is the decisive standard when the model is intended to choose an experimental or clinical action [18,39].

The same standard should be applied to multifunctionality itself. Every added component should have a testable role, an associated CQA, and a control experiment that shows what happens when the component is removed or disabled. A platform with fewer functions but a clear mechanism, reproducible manufacture, and route-specific safety may be more translatable than a highly elaborate nanostructure with impressive in vitro performance. Precision nanomedicine should therefore be understood as the progressive reduction of uncertainty around diagnosis, exposure, and response—not as the progressive accumulation of nanoscale features.

## 13. Conclusions

Multifunctional nanomaterials can connect diagnostics and drug delivery through a common mechanistic sequence: formation and processing create structure; structure creates a property; the property creates a function; and the function must survive biological interfaces, route-specific barriers, product manufacture, and independent validation. AI-assisted plasmonic and terahertz sensors illustrate the difference between model performance and diagnostic evidence. Biopolymers and hydrogels show how interfacial chemistry and matrix structure govern local residence and release. Nose-to-brain systems show that administration route can dominate carrier requirements. Graphene and CNTs reveal how geometry, defects, functionalization, and impurities couple efficacy to safety. Lipid nanoparticles extend the framework to nucleic acid protection, endosomal escape, organ expression, and clinically relevant product definition. Nanoporous, magnetic, and plasmonic platforms add pore-controlled loading, imaging, and actuation.

Across these examples, the decisive question is whether the nanomaterial changes a rate-limiting step that can be measured with an endpoint appropriate to the claim. High internal model accuracy is not clinical validation; high cellular uptake is not necessarily productive delivery; high loading is not bioavailable dose; brain fluorescence is not proof of intact nose-to-brain transport; a slow release curve is not necessarily controlled release; and an imaging signal is not equivalent to active payload exposure. Carrier, payload, and label should be distinguished where their fates can diverge. Safety should be evaluated for the actual derivative, morphology, route, dose, and repeated-use scenario.

A credible future platform will therefore resemble a closed experimental loop: detect a relevant biological state, interpret it with a validated model when appropriate, apply a material or device intervention, quantify the delivered or activated dose, and measure the resulting response. The minimum-evidence roadmap in Table 5 makes the CQA framework operational by requiring material definition, causal functional evidence, payload/release validation, route-specific exposure, biological identity and safety, reproducible manufacture, external/prospective AI validation when used, and comparative translational utility. The opportunity in precision nanomedicine lies not in maximizing the number of functions, but in coupling the minimum necessary functions with enough evidence to reduce uncertainty at each step from concept to product.

## Figures and Tables

**Table 1:** Function-first design variables for diagnostic and drug delivery nanomaterials.

Design Level	Key Variables	What Should Be Measured	Functionality Gate/Common Failure
Material identity	composition; size/shape; porosity; surface chemistry; defects; impurities	orthogonal physicochemical characterization; batch distribution, not only mean values	a nominally identical material behaves differently because morphology or impurity content is uncontrolled
Diagnostic interface	binding chemistry; field confinement; resonance; signal amplification; calibration domain	sensitivity, selectivity, limit of detection, drift, interference, external test performance	high simulated sensitivity without realistic biological validation or independent model testing
Payload state	adsorbed, encapsulated, conjugated, complexed, or precipitated fractions	loading state, free drug, chemical stability, release under relevant media	high total loading but low accessible or pharmacologically active dose
Transport and route	mucus interaction; vascular exposure; tissue penetration; uptake; clearance	route-relevant residence, permeability, biodistribution, target site exposure	optimization in buffer does not survive dilution, mucus, serum, or tissue barriers
Trigger/actuation	pH, redox, enzyme, heat, light, magnetic field, electric field	on/off ratio, trigger threshold, spatial selectivity, tissue-compatible dose	trigger is measurable in vitro but unrealistic or unsafe in vivo
Product definition	stability; sterility; residuals; scale-up; critical quality attributes	release specifications, sensor calibration, particle attributes, storage and process controls	multifunctionality creates too many coupled variables for reproducible manufacturing
Added function/complexity	incremental component; coupling between functions; new impurity and degradation pathways	gain relative to a matched simpler control; mechanism-specific CQA; effect of disabling/removing the added function	additional targeting, imaging, or responsiveness increases complexity without improving a decision, exposure, or outcome

**Table 2:** Representative systems illustrating function-first design lessons across independent and selected case studies.

System/Study	Function	Route/Context	Design Lesson
Graphene-based THz sensor + gradient boosting [5]	dual analyte modeled sensing + prediction	diagnostic design concept	useful for design-space learning, but fabrication and external biological validation remain mandatory
Plasmonic immunosensor image analysis + ML [6]	image-based viral classification	experimental biosensing	paired comparison with LSPR shows what the ML layer adds on the same sensor
Patient-derived plasmonic/SERS platform [8]	disease classification + biomarker inference	clinical biofluid samples	patient samples strengthen relevance, but external/prospective validation remains the next gate
Chitosan systems [9,10]	mucoadhesion, controlled release, wound/local interaction	multiple routes	polymer chemistry should be optimized for the biological interface, not only particle formation
Hybrid nanoparticle–hydrogel depots [11]	local retention + controlled release	local depot	the second component is justified when it solves a distinct retention or release bottleneck
Alginate–GNP/graphene hydrogel [21,49,50]	payload stabilization + release modulation	local/oral-relevant media	a carbon filler is useful only when it changes kinetics, stability, mechanics, or actuation relative to the simpler matrix
Nose-to-brain biopolymer/LGA systems [12,15,44]	route-directed exposure	intranasal/CNS	brain delivery requires PK/biodistribution evidence, not particle size or nasal administration alone
Ionizable LNPs [3,4,16,17]	nucleic acid protection, endosomal escape, organ expression	systemic nucleic acid delivery	composition and degradability must change productive expression, not only particle uptake
Polyamine-coated CNTs carrying microRNAs [22]	nucleic acid delivery + gene regulation	endothelial cells	biological activity of the released RNA is a stronger endpoint than loading or uptake
Morphology-engineered kaolinite/mesoporous silica [34,37,38]	geometry- or pore-controlled loading/release	inorganic local delivery models	accessible surface, pore connectivity, release, and carrier persistence must be measured separately

**Table 3:** Route-specific barriers, misleading surrogate endpoints, and decisive evidence requirements.

Route	Dominant Barriers	Potential Nanomaterial Response	Surrogate to Reject/Decisive Evidence
Intranasal	mucociliary clearance; mucus trapping; enzymatic degradation; low dose volume; regional deposition	mucoadhesive or mucus-penetrating surfaces; stabilization; controlled aerosol/droplet properties	Reject: particle size or brain fluorescence alone. Need: deposition, mucosal safety, repeated-dose tolerance, residence, brain/plasma PK, regional biodistribution, direct-vs.-systemic contribution, carrier vs. payload.
Intravenous	protein corona; complement; liver/spleen uptake; dilution; vascular and cellular barriers	controlled size/surface; stealth or biomimetic design; targeting after systemic fate is understood	Reject: high in vitro cellular uptake. Need: blood stability, corona, complement/hemocompatibility, PK, organ distribution, target site exposure, free vs. carrier-bound payload.
Local/hydrogel depot	diffusion distance; local inflammation; matrix degradation; incomplete release	injectable/implantable network; nanoparticle reinforcement; controlled erosion or local actuation	Reject: high loading or slow mass loss. Need: released species identity/activity, concentration–time profile, mechanism of release, tissue compatibility, degradation/removal, trigger safety.
Wound/topical	exudate; proteases; infection; mechanical disturbance	bioadhesive polymers; antimicrobial surface; moisture-regulating or responsive matrix	Reject: inhibition zone alone. Need: release in wound-like fluid, antimicrobial efficacy with appropriate controls, tissue compatibility, re-epithelialization, irritation/sensitization.
Tumor interventional	heterogeneous perfusion; light penetration; thermal dose; off-target injury	image-guided local devices; plasmonic/magnetic agents; local reservoirs	Reject: image retention or peak temperature alone. Need: spatial dose mapping, optical/thermal dosimetry, drug release/exposure, tumor and normal tissue response, post-treatment monitoring.

**Table 4:** Representative external benchmark systems broadening the design space and translational comparators.

Platform	Primary Function	Key Advance	Critical Question for Translation
Venlafaxine-loaded PLGA nanoparticles, intranasal [44]	brain-directed drug delivery	rapid antidepressant effect in an animal model using a nose-to-brain strategy	how consistently do device deposition and nasal physiology control human brain exposure?
Cationic liposomes for intranasal mRNA [46]	nonviral nucleic acid delivery	brain transfection following intranasal administration	can expression be targeted and repeated without local inflammatory toxicity?
Gold–iron oxide nanoparticles with therapeutic microRNAs [47]	theranostic brain tumor platform	intranasal delivery coupled to multimodal imaging and chemosensitization	which component provides indispensable clinical value, and what is the long-term fate of the inorganic material?
Anti-EphA3 gold–temozolomide nanoparticles [48]	targeted glioblastoma delivery	ligand-targeted intranasal chemotherapy	does receptor targeting increase absolute tumor exposure beyond route effects alone?
Gold nanoparticle–doxorubicin formulation [55]	systemic nanochemotherapy	in vivo assessment of cardiac consequences	nanoparticle incorporation must demonstrate safety rather than assume it
Integrated NIR-II/radionuclide nanotheranostic system [40]	multimodal diagnosis and phototherapy	diagnosis-to-treatment integration with image guidance	can complex synthesis and multimodal instrumentation be standardized at clinical scale?
Closed-loop fiber optic theranostic probe [41]	local sensing + photothermal therapy	pre-, intra-, and post-treatment feedback within one minimally invasive device	how generalizable is the approach across tumor geometries and clinical workflows?
Fiber optic heater + thermoresponsive DOX microgels [42]	on-demand locoregional release	light-to-heat activation with temperature monitoring	can release dose be calibrated in heterogeneous tissues and scaled to clinically relevant lesions?
AI-assisted plasmonic immunosensor [6]	experimental diagnostic classification	ML image analysis improved discrimination relative to LSPR on the same sensor	does performance generalize across devices, operators, matrices, sites, and prospective samples?
Organ-targeted reformulated LNPs [16]	systemic mRNA delivery	composition changed both organ accumulation and productive translation	are targeting, repeated-dose safety, and manufacturing reproducible across species and batches?
BBB-crossing mRNA LNPs [19]	systemic CNS delivery	engineered lipids enabled broad brain cell transfection in preclinical models	does CNS exposure, cell specificity, safety, and expression translate to humans at clinically realistic doses?

**Table 5:** Minimum-evidence roadmap for progression from multifunctional nanomaterial concept to product.

Stage	Minimum Evidence	Key Question	Stop/Go Criterion
1. Material definition	composition; size/shape distribution; surface chemistry; defects/impurities; internal structure or porosity where relevant	what material was actually made, and how does processing create its architecture?	orthogonal characterization defines a reproducible material population
2. Functional mechanism	matched simpler control; perturbation/removal of the added function; formation → structure → property → function chain	does the added feature causally solve a rate-limiting problem?	function changes a mechanistically relevant intermediate endpoint
3. Payload and release	free vs. associated payload; accessible/releasable fraction; chemical/activity assay; mass balance; triggered vs. untriggered control	is active payload delivered by controlled release rather than matrix breakdown or degradation?	bioactive payload and release mechanism are demonstrated
4. Route and exposure	route-specific deposition/residence; PK; biodistribution; target-site exposure; productive intracellular endpoint when needed	does the platform improve exposure through the intended route?	target exposure or productive function improves over matched controls
5. Biological identity and safety	corona/mucus interaction; hemocompatibility or mucosal safety; complement where relevant; degradation/persistence; carrier vs. payload/label fate	is safety evaluated for the actual derivative, route, dose, and repeated-use scenario?	risk profile supports the intended exposure and dose schedule
6. Product/CQA reproducibility	batch distributions; stability; sterility; residuals; device compatibility; process parameters; scale-up stress	can the mechanism-driving attributes be preserved across lots and scale?	predefined CQA acceptance ranges are met across representative lots
7. AI/model validation, if used	locked model/version; independent external data; uncertainty/calibration; prospective manufacture/test or clinical testing	does the model generalize and improve a real design or diagnostic decision?	external validation is passed; prospective validation is passed for decision-guiding use
8. Translational utility	clinically meaningful comparator; benefit vs. added complexity; workflow fit; manufacturability; regulatory measurability	does the multifunctional platform outperform a simpler alternative in a decision-relevant endpoint?	incremental benefit justifies complexity, risk, and product-control burden

## Data Availability

No new data were created or analyzed in this study. Data sharing is not applicable to this article.
